# Ticlopidine to prevent primary arteriovenous fistula failure in hemodialysis patients; a randomized controlled trial

**DOI:** 10.12861/jrip.2013.35

**Published:** 2013-09-01

**Authors:** Ali Ghorbani, Farzad Jasemi-Zergani

**Affiliations:** ^1^Department of Nephrology, Ahvaz Jundishapur University of Medical Sciences, Ahvaz, Iran

**Keywords:** Arteriovenous fistula, Ticlopidine, Hemodialysis

**Implication for health policy/practice/research/medical education:**

Primary arteriovenous fistula (AVF), failure remains a major problem for hemodialysis patients. Vascular access thrombosis prophylaxis needs to start early in the end-stage renal disease patient. Ticlopidine seems to be effective and safe for prevention of primary AVF failure in hemodialysis patients.

## Dear editor–in Chief,

Performance of a successful hemodialysis procedure requires a functional vascular access. The preferred type of access is a native fistula because they have the lowest risk of complications, lowest need for intervention, and the best long-term patency (1). Once an arteriovenous fistula (AVF) is created, it must develop to the point that it is usable. Vascular access dysfunction is one of the most important causes of morbidity in the hemodialysis population (2). Primary failure of native fistulas occurs as a result of either thrombosis within the first several weeks following surgical creation (early thrombosis), or inadequate maturation of the vein (3). The primary AVF failure rate is approximately 9-50% (4-6). Fistula evaluation 4–6 weeks after creation should be considered mandatory (7). The clinical manifestations of early fistula failure are failure to develop adequately to permit repetitive cannulation for dialysis, inadequate flow to support dialysis, and thrombosis. The characteristic pathology that results in AVF failure is a juxta-anastomotic stenosis (8). Whether primary AVF failure can be prevented with pharmacologic agents has not been extensively examined. Several studies have indicated that the frequency of AVF failure and loss can be reduced with antiplatelet agents (9-18). Although those results are encouraging, they do not provide conclusive evidence of the efficacy of antiplatelet agents among patients with AVF. In the present study, we performed a randomized, double-blind trial to test the hypothesis that ticlopidine, would prevent primary AVF failure among hemodialysis patients with AVF. The study was a randomized, double-blind trial. Patients eligible for enrollment in the study included at least 18 years age patients close to the initiation of chronic hemodialysis who required AVF and patients who were undergoing chronic hemodialysis but required a new AVF at a different site. Exclusion criteria included patients with a history of gastrointestinal bleeding or previous bleeding episodes about 6 months prior to initiation of study, patients already receiving chronic anticoagulation therapy (antiplatelet agents or warfarin), patients with terminal or life-threatening disease, pregnancy, or malignant hypertension, a platelet count of <100,000 /µL or known coagulation abnormalities and demonstrated other medical conditions that would make antiplatelet therapy dangerous. All patients were recruited from the outpatient hemodialysis program at Jundishapur University, and the same surgical team placed all fistulas. Randomization was performed centrally, by the coordinating center. The randomization was stratified according to medical center with a permuted block scheme, with a block size of four and equal allocation. After identifying and obtaining consent from eligible participants, the local study coordinator telephoned the coordinating center to obtain a randomization number, which corresponded to a specific medication bottle available in the local research pharmacy. Neither the details of the randomization sequence nor the identity of the medication assignment was known to the participant or any personnel at the participating sites. Consenting eligible participants were randomized to receive either ticlopidine (250 mg twice daily) or matching placebo (32 patients in each group). The treatment was initiated 7-10 days prior to scheduled access surgery and continuing for 8 weeks postoperatively. The primary null hypothesis of the study was that ticlopidine would have no preventive effect on the incidence of primary AVF failure. The primary outcome was unassisted fistula patency 8 weeks after fistula creation. Fistula patency was determined by a member of the study team (either the study coordinator or the site principal investigator), who was blinded to treatment allocation. The fistula was classified as patent if a bruit was detectable along the vein at least 8 cm proximal to the arteriovenous anastomosis throughout systole and diastole. The major secondary outcome was fistula suitability for dialysis. Fistula suitability is defined as the ability to use the fistula for dialysis during the period 180 days after fistula creation, and obtain a minimal dialysis machine blood flow of 200 mL/min. Other secondary outcomes included adverse events, and mortality. Platelet hemostatic function was measured monthly, as whole-blood bleeding time. Routine blood chemistry profiles, dialysis prescriptions, body weights, medications, and complications for all patients were recorded in a computerized database and thus were available for inclusion in the final data analysis. We obtained detailed information on bleeding events. Assessment of the severity of bleeding episodes was performed by a panel blinded to the treatment assignments. Discontinuation of study drug after any bleeding events was left to the discretion of the participant’s primary nephrologist. (1) The research followed the tenets of the Declaration of Helsinki; (2) informed consent was obtained; (3) the research was approved by ethical committee of Jundishapur University of Medical Sciences. All data were tested for normality using the method of Kolmogorov-Smirnov. Statistical analysis were performed on an intention-to-treat basis. The t-test was used when the means of two groups were compared. The cumulative incidence of the AVF failure was estimated with the Kaplan-Meier method, and differences in rates between treatment groups were tested with the log rank test. The cumulative incidence of the first bleeding event was analyzed in a similar manner. On the basis of intention-to-treat principles, all other participants for whom study medications were discontinued continued to be monitored according to the protocol. All hypothesis tests were conducted by using a significance level of 0.05 (two-sided). The SPSS 13 (SPSS Inc., Chicago, IL, USA) was used to analyze the data. Between December 2004 and June 2005, a total of 64 patients met the study criteria for enrollment. There were no significant differences in age, body mass index (BMI), gender, cause of renal failure or time to initiation of fistula use between the ticlopidine-treated and control groups. All patients were taking study medication 3-7 days prior to surgery. Two patients in ticlopidine group and three patients in placebo group were enrolled after creation of a second fistula. Two patients died before the end of trial. None patient was lost to follow-up. Five vascular accesses in ticlopidine-treated group and three fistulas in placebo group were proximal AVF. Two patients in ticlopidine-treated group showed an early failure of the AVF compared to nine patients in placebo group (p< 0.05). Therefore, the primary AVF failure rate at the end of the study period was 29% among patients who received placebo versus 6.45% among patients who received ticlopidine. There was significant benefit of active treatment in the prevention of AVF failure. The hazard ratio for the incidence of primary AVF failure was 0.22 (CI 95%, 0.2 – 1.01). The Cox proportional-hazards regression model was used to analyze the association of several co-variables with the development of primary AVF failure. None of the co-variables measured at baseline or follow-up times was correlated with the development of AVF failure. Fistula locations had no effects on the development of AVF failure in either group. Hemodialysis was initiated in 26 patients of ticlopidine group within six months after AVF creation. Among them hemodialysis was successfully done in 24 patients. In placebo group, hemodialysis was initiated in 20 patients successfully. Three patients in ticlopidine group and two patients in placebo group did not undergo hemodialysis after six months. There was no statistically significant difference in the suitability of AVF for hemodialysis between groups. The cumulative incidence of bleeding was similar between groups. Three patients in ticlopidine group and one patient in placebo group experienced mild bleeding. No severe bleeding episode was recorded during active treatment period. There were no deaths attributable to bleeding in either treatment group. Bleeding times were similar at baseline and remained stable throughout the study period. In addition, there were no differences between baseline and follow-up hematocrit values or changes in recombinant human erythropoietin doses during the study period for either group. Chronic maintenance hemodialysis requires stable and repetitive access to the intravascular compartment in order to deliver high rates of blood flow to the extracorporeal circuit. The AVF is the method of choice for the establishment of hemodialysis vascular access in patients with end-stage renal disease. The fistula is relatively simple to perform under local anesthesia and, when successfully established, is easy to needle and relatively free from complications. However, a significant proportion (9-50%) of fistulas fails early within 3 months of surgery (4-6). An AVF with primary failure is defined as a fistula that never provided reliable hemodialysis (18). Vascular access failure is the most common reason for hospitalization among hemodialysis patients (19). The typical lesion of access thrombosis is neointimal vascular smooth muscle cell proliferation in the anastomotic draining vein. Platelet activation from endothelial injury may play an important role in stimulating platelet aggregators such as PDGF and thromboxane A2, in addition to directly stimulating vascular intimal proliferation (18). Therefore the therapeutic potential of antiplatelet agents Including aspirin, sulfinpyrazone, dipyridamole, and ticlopidine were tested (9-17). Our study was undertaken to determine the effects of ticlopidine on the incidence of primary AVF failure among newly created AVFs. We observed a significant risk reduction in the primary AVF failure in active treatment group compared to placebo group. The results of our analysis suggest that twice daily administration of 250 mg of ticlopidine, beginning 7-10 days prior to AVF creation, was successful in preventing the development of vascular failure with acceptable side effects. Using a proportional-hazards regression model, we were unable to account for the differences observed in our clinical trial on the basis of age, gender, diabetes mellitus, race, baseline or follow-up lipid profiles, bleeding times, hematocrit levels, or weekly doses of recombinant human erythropoietin. This finding suggests that the risk reduction in vascular failure might be attributed to ticlopidine administration. Our results are supported by recent Cochrane report (20). This meta-analysis confirmed the beneficial effect of antiplatelet treatment as an adjuvant to increase the patency of A-V fistulae in the short term. However, there have been multiple studies showing variable results of antiplatelet agents on vascular access failure. Yevzlin *et al*. showed a negative association between antiplatelet therapy and access patency (21). In this trial there were no drugs associated with significant risk reduction in access failure. Moreover, in patients with access failure, antiplatelet agents were associated with increased risk of access failure. Kaufman *et al*. demonstrated no change in the risk of graft thrombosis with aspirin plus clopidogrel therapy (22). They also noted that in chronic HD patients, aspirin therapy was associated with increased risk of thrombosis. Also Kooistra *et al*. were unable to demonstrate a benefit with low-dose aspirin on thrombovascular events in 68 hemodialysis patients (23).In contrast combining all the studies of antiplatelet agents in patients with new primary fistulae in which there was a placebo control group, the thrombosis rate in the control group was significantly higher than active treatment group (20). Three trials compared ticlopidine with placebo with a total number of 312 participants undergoing A-V fistula formation or graft interposition. All three trials comparing ticlopidine with placebo favored treatment. In the Fiskerstrand study, two out of six patients in the ticlopidine group compared with five out of nine in the placebo group, developed fistulae thrombosis at one month (OR = 0.40, CI 95%, 0.05-3.42) (15). In the earlier Grontoft study, only two out of 19 who received treatment developed fistulae thrombosis compared to eight out of 17 on placebo (OR = 0.13, CI 95%, 0.02-0.76) (13). In Gontoft 1998, 16 out of 130 patients who received ticlopidine developed thrombosis in the fistulae compared with 25 out of 131 in the placebo group (OR = 0.60, CI 95%, 0.30-1.18) (14). The overall result of the meta-analysis also favored treatment (OR = 0.47, CI 95%, 0.26-0.85). The overall p was 0.01 (20). We also assessed the effect of ticlopidine on the successfully initiation of hemodialysis via AVF. The rate of the performing successful first hemodialysis via AVF was similar between groups despite difference in AVF failure. This finding might be related to limited number of subjects in our trial. Though, we recommend assessing this item in another trial with sufficient number of subject. The overall incidence of bleeding events was 6.25% in our study. According to Kaufman *et al*., we expected to encounter with 16 episodes of bleeding during 6 months in our patients (22). However, the incidence of bleeding episodes was lower than expected. This finding might be related to restrictive exclusion criteria. This study is the first carefully monitored trial that was limited to AV fistula. It is a well- known fact that AVFs have lower thrombosis rates compared with arteriovenous grafts. In multiple studies, AVFs have been shown to have significantly improved patency rates and lower complication and infection rates. Some studies pointed out that antiplatelet therapy might be more effective in fistulas than in arteriovenous grafts (14,22). Our results suggested that ticlopidine is an effective preventive therapy in primary AVF failure. The major limitation of our study is a small number of patients, but in view of the promising results we believe that our preliminary findings deserved prompt communication. However, the data must be interpreted with caution because the pharmacological approach to prevent vascular access thrombosis in hemodialysis is still in its infancy. Overall, the effect of antiplatelet agents on vascular access patency needs further investigation. A prospective randomized controlled trial including a larger number of patients is warranted. Currently the national institutes of health- sponsored dialysis access consortium are conducting an ongoing double-blind multicenter randomized evaluation of clopidogrel in AVF patency (24). Primary AVF failure remains a major problem for hemodialysis patients. Vascular access thrombosis prophylaxis needs to start early in the end-stage renal disease patient. Ticlopidine, beginning 7-10 days prior to AVF creation and continuing for 2 months, seems to prevent primary AVF failure with acceptable side effects in selected hemodialysis patients.

## Authors’ Contributions

AG defined the aims of research. AG and FJZ prepared the paper. AG edited the manuscript and prepared the final manuscript.

## Conflict of Interests

The authors declare that they have no conflict of interest.

## Ethical considerations

Ethical issues (including plagiarism, data fabrication, double publication) have been completely observed by the author.

## Funding/Support

This work was financially supported by a grant from vice chancellor for research of Ahvaz Jundishapur University of Medical Sciences.
